# Towards a Mechanism for Poly(I·C) Antiviral Priming in Oysters

**DOI:** 10.1128/mBio.00407-20

**Published:** 2020-03-24

**Authors:** Nelson E. Martins

**Affiliations:** a Université de Strasbourg, CNRS UPR9022, Strasbourg, France

**Keywords:** ostreid herpesvirus, interferons, invertebrate-microbe interactions, mollusks

## Abstract

Viral diseases cause significant losses in aquaculture. Prophylactic measures, such as immune priming, are promising control strategies. Treatment of the Pacific oyster (Crassostrea gigas) with the double-stranded RNA analog poly(I·C) confers long-term protection against infection with ostreid herpesvirus 1, the causative agent of Pacific oyster mortality syndrome. In a recent article in *mBio*, Lafont and coauthors (M. Lafont, A. Vergnes, J. Vidal-Dupiol, J. de Lorgeril, et al., mBio 11:e02777-19, 2020, https://doi.org/10.1128/mBio.02777-19) characterized the transcriptome of oysters treated with poly(I·C). This immune stimulator induced genes related to the interferon and apoptosis pathways. This response overlaps the response to viral infection, and high expression levels of potential effector genes are maintained for up to 4 months. This work opens the door to characterization of the phenomena of immune priming in a poorly studied invertebrate model. It also highlights the importance of interferon-like responses for invertebrate antiviral immunity.

## Abstract (precis)

Viral diseases cause significant losses in aquaculture. Prophylactic measures, such as immune priming, are promising control strategies. Treatment of the Pacific oyster (Crassostrea gigas) with the double-stranded RNA analog poly(I·C) confers long-term protection against infection with ostreid herpesvirus 1, the causative agent of Pacific oyster mortality syndrome. In a recent article in *mBio*, Lafont and coauthors (M. Lafont, A. Vergnes, J. Vidal-Dupiol, J.

## COMMENTARY

Viral infectious diseases cause significant economic losses to aquaculture. While bacterial infections are commonly controlled by the use of antibiotics, we still lack effective antivirals. Viruses are obligate intracellular parasites, offering few targets for specific inhibitors. As a consequence, prophylaxis is the most effective way to control viral infections. In mammals and other vertebrates, prophylactic strategies against viruses are based on specific priming of the adaptive immune systems by vaccination. However, invertebrates do not have the adaptive arm of the immune system, relying solely on innate immunity. The development of prophylactic measures in economically important invertebrate species requires a better understanding of their antiviral defenses.

Innate immunity is the first line of defense against viruses in all animals. In vertebrates, the production of interferon (IFN) family cytokines is the hallmark of the innate antiviral response. These are produced by virus-infected cells upon recognition of different pathogen-associated molecular patterns (PAMPs), such as double-stranded RNA (dsRNA), a hallmark of RNA virus replication or convergent transcription in DNA viruses. dsRNAs are recognized by specialized receptors such as Toll-like receptor 3 (TLR3), melanoma differentiation-associated protein 5 (MDA5), or retinoic acid-inducible gene I (RIG-I), depending on dsRNA length, the presence of 5′ triphosphates in the extremities, and intracellular localization. Recognition of dsRNA triggers intracellular cascades that culminate in the translocation to the nucleus of interferon regulatory factors (IRF), among other transcription factors. This results in the production of interferons and induction of a large array of antiviral genes to control the infection.

**FIG 1:**
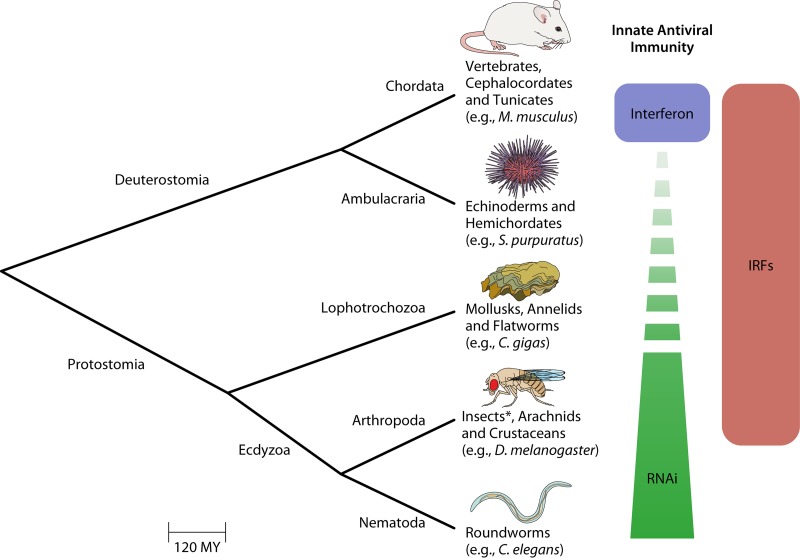
Simplified metazoan taxonomy of the major animal clades discussed in the text. Invertebrates are not monophyletic, comprising both deuterostomes (e.g., sea urchins) and protostomes. Innate antiviral defenses are defined mainly for mammalian vertebrates (Chordata), insects (Arthropoda), and roundworms (Nematoda). The production of interferons (IFN), controlled by interferon regulatory factors (IRFs), among other transcription factors, is the hallmark of the vertebrate innate antiviral immunity. Constitutive RNA interference (RNAi) is described as the major antiviral defense in invertebrates but has mainly been characterized in ecdysozoa. Inducible responses are poorly described outside vertebrates. Multiple invertebrate clades (Ambulacraria, Lophotrochozoa, and some arthropods) have homologs for IRFs which may play a role in their antiviral response. IRFs were lost in insects (*) and nematodes. The work by Lafont et al. (5) identified an IFN-like response in the mollusk *Crassostrea gigas*. The scale bar is in million years (MY).

In animals devoid of interferons, such as invertebrates, RNA interference (RNAi) is often described as the major antiviral defense (Fig. 1) (1). Since antiviral RNAi is shared with distantly related eukaryotes (e.g., plants), it is postulated that the interferon response replaced the antiviral function of RNAi in vertebrates (2). However, most experimental work supporting this assumption comes from two main models, the insect Drosophila melanogaster and the nematode Caenorhabditis elegans, two ecdysozoan protostomes. Ecdysozoa, together with the Lophotrochozoa (mollusks and annelids) and the Platyzoa (e.g., flatworms), form the major subdivisions of the protostomes. Ecdysozoa have repeatedly lost IRFs (3); hence, they are not fully representative of the diversity of immune pathways potentially present in protostomes. Other protostomes, including mollusks, not only have multiple IRF genes (3) but also homologs of several other components of the of the IFN pathway, including sensors (MDA5, RIG-1, and TLRs), signal transducers (MAVS, STING, MyD88, and IκKB), transcription factors (NF-κB/Rel), and effectors (OAS, Viperin, and Mx) (4).

The work directed by Caroline Montagnani (5) sheds new light on the importance of IFN-like responses for invertebrate antiviral immunity. The authors and others had previously shown that treating oysters with dsRNA or poly(I·C) confers a long-term protection against ostreid herpesvirus 1 (OsHV-1) (6). This virus causes Pacific oyster mortality syndrome, which represents an important economic burden for shellfish farmers. Using RNA sequencing, Lafont et al. explored the genome-wide transcriptomic changes triggered by poly(I·C), a synthetic dsRNA analog that is a potent agonist of TLR3 and MDA5 in mammals and of OsHV-1 (5). Poly(I·C) treatment induced a strong transcriptional response (more than 4,000 genes differentially expressed, representing approximately 16% of the annotated transcriptome), with a majority of genes upregulated at one or several time points posttreatment. Most interestingly, the majority of genes induced by poly(I·C) are also induced by OsHV-1 infection, suggesting that poly(I·C) primes an immune response similar to that of the virus, which results in a protective antiviral state. Further supporting this interpretation, Lafont et al. observed only minor transcriptional changes following OsHV-1 infection in poly(I·C)-primed oysters (5). Strikingly, gene ontology analysis revealed a strong enrichment of immune functions among poly(I·C)-induced genes. Many of these genes belong to known antiviral pathways characterized in mammals, such as the IFN (e.g., MDA5, STING, OAS, and Viperin) and apoptosis pathways. If a role of IRF transcription factors in the regulation of these genes can be confirmed, this would suggest that the IFN pathway in vertebrates was grafted to an ancestral antiviral response regulating a repertoire of evolutionarily ancient restriction factors.

Remarkably, the authors show that the induction of several potential antiviral effectors is sustained for several days or even months after stimulation with poly(I·C). This sustained induction of an antiviral response is the possible underlying cause of the observed immune priming. Indeed, innate immune priming and memory have been described in several species, including invertebrates that lack an adaptive immune system. However, the molecular mechanisms are poorly characterized and are probably of a heterogeneous nature (7). The findings reported here provide an interesting model to start to functionally explore the molecular mechanisms at play in immune priming of a long-lasting response.

In light of the potential use of priming with poly(I·C) as a prophylactic strategy in oysters (referred to as “pseudovaccination” by the authors), a question remains pertaining to the potential impact of a persistent expression of antiviral genes on oyster physiology, which may impact productivity. Indeed, even though the authors did not observe an impact of poly(I·C) treatment alone in the survival of oysters (6), sustained expression of interferon-stimulated genes (ISGs) in humans is associated with a number of diseases known as interferonopathies (8).

Several additional questions remain open in this fascinating model. How are the viral genetic material and poly(I·C) recognized? The oyster RIG-I homolog is able to bind poly(I·C) *in vitro* (9), so this is a main candidate for a receptor of dsRNAs. Nevertheless, like in vertebrates, there could be specialized receptors for different PAMPs, and the roles of the multiple TLRs and of the cGAS/STING orthologs remain to be characterized. Would agonists of these pathways yield the same protective effect? The effector mechanisms also remain to be identified. A first interesting point to address would be to determine how broad the protection conferred by poly(I·C) is by infecting oysters with other families of viruses. This would imply an effort to explore molluscan viruses, a poorly developed field that deserves further attention. In humans, the functional characterization of hundreds of interferon-stimulated genes already has revealed several novel antiviral effectors (10). Others demonstrated that while the core interferome of vertebrates contains the best-characterized antiviral proteins, each clade has several specific ISGs whose antiviral function is unknown (11). Given the diversity of invertebrate species, their antiviral defenses constitute an unexploited reservoir of novel antiviral genes with potential relevance to human health.
